# Implementation and proficiency analysis of enhanced graph algorithm for DC microgrid applications

**DOI:** 10.1038/s41598-024-65225-8

**Published:** 2024-06-24

**Authors:** Mohamed Abdullah J, Sumathi V

**Affiliations:** 1 School of Electrical Engineering, Vellore Institute of Technology, Chennai, Tamil Nadu India; 2 Centre for Automation, School of Electrical Engineering, Vellore Institute of Technology, Chennai, Tamil Nadu India

**Keywords:** Energy science and technology, Engineering

## Abstract

Integrating renewable energy generation with the conventional grid supports reduces carbon emissions in the atmosphere. Despite technical advancements in protection strategies, critical issues concerning renewable integration in microgrid structures require standardized solutions. The essential aspects that need to be concentrated during securing the grids are rapid fault interruption, false tripping and blinding of protection. This study proposes an innovative approach to enhance fault isolation speed through the implementation of a grid monitoring system (GMS) coupled with a fault identification method based on Kosaraju’s algorithm. This algorithm operates on the principles of overvoltage and overcurrent detection. The study assesses the efficacy of this approach by examining its integration with a Z-source circuit breaker and conducting tests on different fault types within a 13-bus system. Real-time simulations using Opal RT software are employed to experimentally validate the proposed methodology, ensuring its efficacy in fault interruption and isolation.

## Introduction

The rise in demand for DC loads in residential, commercial, and industrial applications has a predominant effect on including renewable energy sources (RES) in the current power system. DC microgrids outperform AC microgrids in dependability, flexibility, efficiency, and resilience since there are no power factor issues or frequency-related issues as in AC microgrids. Though the DC microgrid has been proven more efficient, a sensitive and rapid protection mechanism is necessary to ensure its reliability. Due to the non-alternating nature and fast dynamics of DC current, the protection of the DC microgrid is one of the main concern, which includes fault current level, false tripping, protection blinding, auto reclosing prohibition, and unsynchronized reclosing. Bidirectional protective devices have become the solution to protect the power electronic devices and renewables connected to the grid from earlier issues. The DC Circuit breaker (DCCB) topologies suppress the faults rapidly and effectively handle the fault in a system with no influence on the integrated renewables^1^.

It is essential to understand the DC microgrid classification while designing an effective protection scheme incorporated with adaptive protective schemes for the DC network. DC bus microgrid is classified as a Unipolar and Bipolar polarity configuration. The Unipolar DC bus microgrid system^2^ contains only two polarities, positive and negative bus voltage, to carry supply to the load. In contrast, the Bipolar DC bus microgrid system^3,4^ provides three different voltage (+ Vdc, −Vdc and 2Vdc) options to the utility connected in the microgrid, which comes under polarity-based architecture. The Bipolar DC bus is more flexible in securing the load and more resourceful for switching the load to secured polarity during fault conditions by providing various voltage sources. However, the difference in load distribution creates an unbalanced system to overcoming this issue an additional voltage balancer circuit in the DC bus system is required.

Single bus DC microgrid, Multibus DC microgrid and Reconfigurable topology are classifications based on network topologies used in DC microgrid. The most commonly used topology in DC microgrids is the single-bus topology the source, load and battery are all connected in the typical single-bus system. The Multibus DC microgrid system^5,6^ comprises several microgrids connected in series or parallel to provide power-sharing capability within individual microgrids. Reconfigurable topology is an innovative solution for achieving a flexible microgrid system using intelligent electronic devices (IED) during contingencies .

During maintenance or fault interference in a microgrid, the system reconfigures itself into a Ring-type^7^ or multi-terminal type topology^8^, depending upon the required power flow paths. The ring-type topology is adapted for bidirectional power flow, whereas the multi-terminal type is adapted for more reliable multiple power flow paths^9^. Apart from their flexible and dependable nature, the reconfigurable type topology is complicated and has power limitations compared to the conventional topology.

The interfacing methods provide different configurations for interfacing the AC grid with the DC microgrid system for grid integration. Radial configuration is one of the conventional methods of interfacing AC and DC microgrid systems that provides a way for power flow to supply the loads, and the bus configuration varies depending on user requirements^10, 11^. This configuration is commonly used in LVDC to provide residential loads and enables power sharing between neighbouring buses. Since this configuration has a radial structure, the reliability of a single bus system is still being determined, as a single fault that occurs will blackout the entire bus system. If the system is a multi-bus radial configuration system, it can isolate the faulted bus and provide flexible power-sharing between the healthy buses.

Ring configuration type AC interfacing^12^ allows two or more power flow paths to the load; by deploying IED, the system stands to be more reliable and flexible than radial configuration. The role of the IED is to monitor and interface each bus, isolate fault buses using fast-acting DC switches and ensure the supply to other buses until the fault is cleared. This configuration relies majorly on AC grid supply, so the supply from the utility to the DC bus microgrid is completely interrupted during an AC feeder fault. Two or more sources from the AC grid are used in the interconnected configuration; the two types of interconnected configuration are Mesh and Zonal. Mesh-type interconnected configurations allow more than one AC microgrid to interface with the system, and they are widely used in HVDC distribution applications. Zonal-type connected configurations divide the distribution system into different zones, and each zone contains additional DC buses connected with the AC utility and distributed energy resources. Both types are more reliable and flexible for switching or isolating faults and can perform multiple islanding operations. However, these types of interconnected configurations are more complicated in design and function, requires various coupling interfaces.

In the above-described configuration, the application of the protection strategy is complicated because of fundamental issues such as the absence of zero crossing, as in AC systems. The protection scheme implemented in these configurations uses one type of relay for all locations where the relays cannot identify the difference in forward and reverse fault conditions. So, implementing different relays at different locations based on the purpose of the requirement is mandatory. Also, fault prediction is more linear than other protection schemes in the DC microgrid system if zone-based protection with an adaptive algorithm using the overcurrent method is implemented to avoid false tripping and unsynchronised reclosing of breakers. So, selecting an algorithm based on the above factors, such as microgrid operating conditions, topology, distributed energy resources, modes of operation and fault current contribution, is an essential aspect of protection strategy in DC microgrid. Hence, adaptive protection schemes prioritize algorithms capable of adjusting relay settings according to the system's stability. These adaptive settings are either recalculated and programmed into the relay following a significant change in conditions or pre-configured as multiple group settings derived from previously computed scenarios. This approach addresses the classic adaptive overcurrent protection problem, ensuring a dynamic response to evolving system dynamics.

In ^13^, a rapid protection method based on fault current polarity has been suggested. However, this method could be more precise and distinguish between internal and external faults for fault resistance values greater than 1. Certain algorithm-based protection techniques predict the active bus and identify the minimal distance from the fault occured region to the nearest distributed generation (DG). These algorithms act faster than others as they use data structure and graph theory techniques for fault detection in DC microgrids^14–16^. As the protection system for DCMG is more dependent on the configuration, an appropriate protection scheme should be designed which is adaptable for all system configurations. So, this proposal suggests a fault zone detection technique using a Kosaraju algorithm using Python scripts in the protection controller, and a smart power relay (SPR)^17^ is integrated based on the location and purpose of implementation. The SPR can be implemented with bipartite matching algorithm to fix the relay threshold settings and the values are stored in heap. The effectiveness of the proposed work is proved with bidirectional Z-source DCCB integrated with graph algorithm which improves rapid fault current interruption providing durability over time^18^. Solid state circuit breaker (SSCB) due to their intricate circuitries, necessitate more complex control schemes and entail higher costs^19^. They typically incur higher on-state losses and possess a lower interruptible fault current in comparison to mechanical and hybrid CB. While the on-state losses of SSCBs can be mitigated by utilizing wide band gap devices, a Z-SSCB stands out by eliminating the necessity for additional control or detection circuitry once its triggering conditions are satisfied. It boasts a simpler topology, faster response time, higher energy density, and a reduced price compared to other types of CBs. While mechanical and hybrid CBs find suitability in high voltage transmission scenarios, Z-SSCBs excel in applications involving medium and low voltages. Numerous investigation considering system uncertainty, computational latency and several studies examining the performance of fault detection for different faults applied across pole-pole (P-P), pole-ground (P-G) and double pole-ground (PP-G) is compared and elaborated in Table 1. On comparison, the enactment of pseudo scheme is quiet deliberate compared to other techniques. Fault response with ultra-fast detection is a popular choice as the presence of grid assets are more essential.

**Table 1:** Performance comparison of different fault detection algorithm under various conditions.

Fault diagnosis method	Mathematical morphology algorithm ^20^	Discrete median filter & mathematical morphology algorithm ^21,22^	Distance method ^23^	Extended kalman filtering algorithm ^24^	Pseudo data driven based scheme ^25^	ANN-based scheme ^26^	Entropy-based scheme ^27^
Types of fault analysed	P-P, P-G	P-P, PP-G, NP-G	P-P, PP-G, P-G	P-P, PP-G, NP-G	P-P, PP-G	P-P, P-G	P-P, P-G
Fault clearing time	5 ms	2.5 ms	1.8–2.4 ms	1 ms	75000 ms	1 ms	0.2 ms
Noise	Not analysed	Analysed	Analysed	Not analysed	Not analysed	Analysed	Not analysed
Response to fault	Fast	Ultra-fast	Fast	Ultra-fast	Slow	Fast	Ultra-fast
Breaker type	SSCB	SSCB	IED	SSCB	SSCB	SSCB	SSCB
Test system topology	Radial	Radial, mesh, loop	Zonal	Mesh	Mesh	Mesh	Radial, loop
Uncertainty	Varying fault resistance	DG dynamics, fault resistance, change in grid configuration	Varying fault resistance	Varying fault resistance	DG dynamics, fault resistance	Varying fault resistance	High impedance fault
Reclosing time	–	–	10 ms	0.35 ms	–	3 ms	1 ms
Efficiency in %	95.5%	99.5%	95%	99.9%	97.5%	99.38%	99.9%
Computational latency	Low	Low	Low	Very low	Very high	High	High
Configuration	MVDC	MVDC	LVDC, MVDC	MVDC	LVDC	HVDC	MVDC

The function of bipartite matching involves operating within a graph or network, either as cohesive or separate sets. In a ring configuration, it operates within cohesive sets, whereas in a radial configuration, it operates within separate sets. It's important to note that GABPC oversees and adjusts relay settings, ensuring they adhere to voltage, current, and power thresholds within a range of ± 12% of rated values. Expanding on bipartite matching, Kosaraju’s algorithm can be employed to safeguard a DC microgrid system by identifying functional and disconnected buses. This algorithm identifies strongly connected components (SCCs), which represent subsets of vertices in a directed graph and, in the context of a microgrid, correspond to functional bus nodes. Initially, the algorithm conducts a Depth-First Search (DFS) within the microgrid system, pinpointing active and inactive nodes in both forward and reverse directions. This meticulous process allows for prompt identification of faults and transients without undue delay. Additionally, Dial’s algorithm plays a crucial role in determining the shortest path for a fault to travel from its origin to the nearest DG, enabling the tripping of the breaker closest to the fault location.

As the proposed fault detection technique works bi-directionally, the reverse fault current detection and interruption and the efficacy of the proposed solution will be proven through experimental results obtained using an Opal Real-Time Simulator. The intended outcome of this paper is to analyse all challenges, particularly those related to protecting the DC microgrid using algorithm-based protection for system modelling and data analysis. The paper's organization is as follows: Section II briefly describes the modelling of a nine-bus DC microgrid system and Z-source breaker topologies. Section III explains fault detection using the Kosaraju’s algorithm with the overcurrent detection principle. Section IV discusses the proposed work's results. Section V briefs the conclusion and future works.

## Modelling of thirteen bus DC microgrid system

An isolated/autonomous microgrid operates exclusively, where each unit in the microgrid connects to a 600 V DC bus. The DC link capacitor controls the DC voltage and keeps it steady at the DC bus. The algorithm based adaptive protection supports protection for LVDC and MVDC system with range upto 5 kV. In this article a 600 V, 30 kW system with renewable resources considered includes a 3kWh battery, a 15 kW Photovoltaic module, and a 12 kW Permanent Magnet Synchronous Generator (PMSG)-based wind turbine.

The integration of a Photovoltaic (PV) array into a DC microgrid enhances real-time system performance. Incorporating PV panels in series increases voltage, while parallel-connected PV panels boost current output. With a solar irradiance level of 1000W/m^2^, the PV module design is grounded on the Shockley diode equation.

Considering V_pv_ = V_oc_ , N_p_ = 1 and N_s_ = 30, the PV module is designed based on the given equation for I_PV_ which can be denoted as,

1 $$I_{PV} = I_{Ph} - I_{O} \left[ {\exp \exp \left\{ {\frac{{q \times (V_{PV} + I_{PV} R_{S} )}}{{N_{S} AKT}}} \right\} - 1} \right]$$

where I_ph_ is the photo-current of a module, I_rs_ is the reverse saturation current of a module, I_PV_ is the output current of a PV module, I_o_ is the saturation current of a module which varies with temperature of a cell, number of cells connected in series is denoted as N_s_ and R_s_ is series resistance respectively. Depending on the voltage and current requirement, the proposed PV array consists of 31 PV modules integrated to extract an output voltage of 600 V for a standard solar irradiance with cell temperature maintained at 1000W/m^2^ and 25 °C respectively^28^. Furthermore, the boost converter interfaced with the photo-voltaic array to increase the required voltage levels.

In a PMSG, the permanent magnet in the rotor provides the field excitation. Considering a fixed speed wind turbine with a wind speed taken as 72 m/s and zero pitch angle is 0°, the aerodynamic model of a wind turbine is given by

2 $$P = {1}/{2}\rho AC(\lambda )\nu^{{3}}$$

where P is the wind power generated, ρ is air density, A is the area covered by the blades, v is the wind speed, Cp is the power coefficient and λ is tip speed ratio.

The tip speed ratio of a wind turbine is given by

3 $$\lambda = Rn\pi /30\nu$$

where n is wind turbine rotor speed in revolutions per minute(r/min).

4 $$T_{m \, } = P_{\omega } /\omega_{m} = \, \left[ {0.5\rho \, A_{r} C_{p} \left( {\alpha , \, \beta } \right) \, v3} \right] \, /\omega a_{m}$$

The fixed-speed PMSG wind turbine is designed to produce an output of 600 V. The torque generated by the wind turbine is then directed to the PMSM generator, which converts the mechanical energy to 3-phase electrical energy. A rectifier is interfaced to convert the AC input from the turbine to DC output with an LC filter with values of 47 and 220 µF, respectively^29^.

Battery modelling is of adequate importance since the microgrid has a bidirectional power flow. So, bidirectional battery modelling with a buck-boost converter is required to interface with the DC bus^30^. The overall DC system is modelled as one point grounding to analyse the proposed strategy under various operating condition.

## Modelling of bidirectional DC circuit breaker

### Response during steady state operation

**Figure 1:**
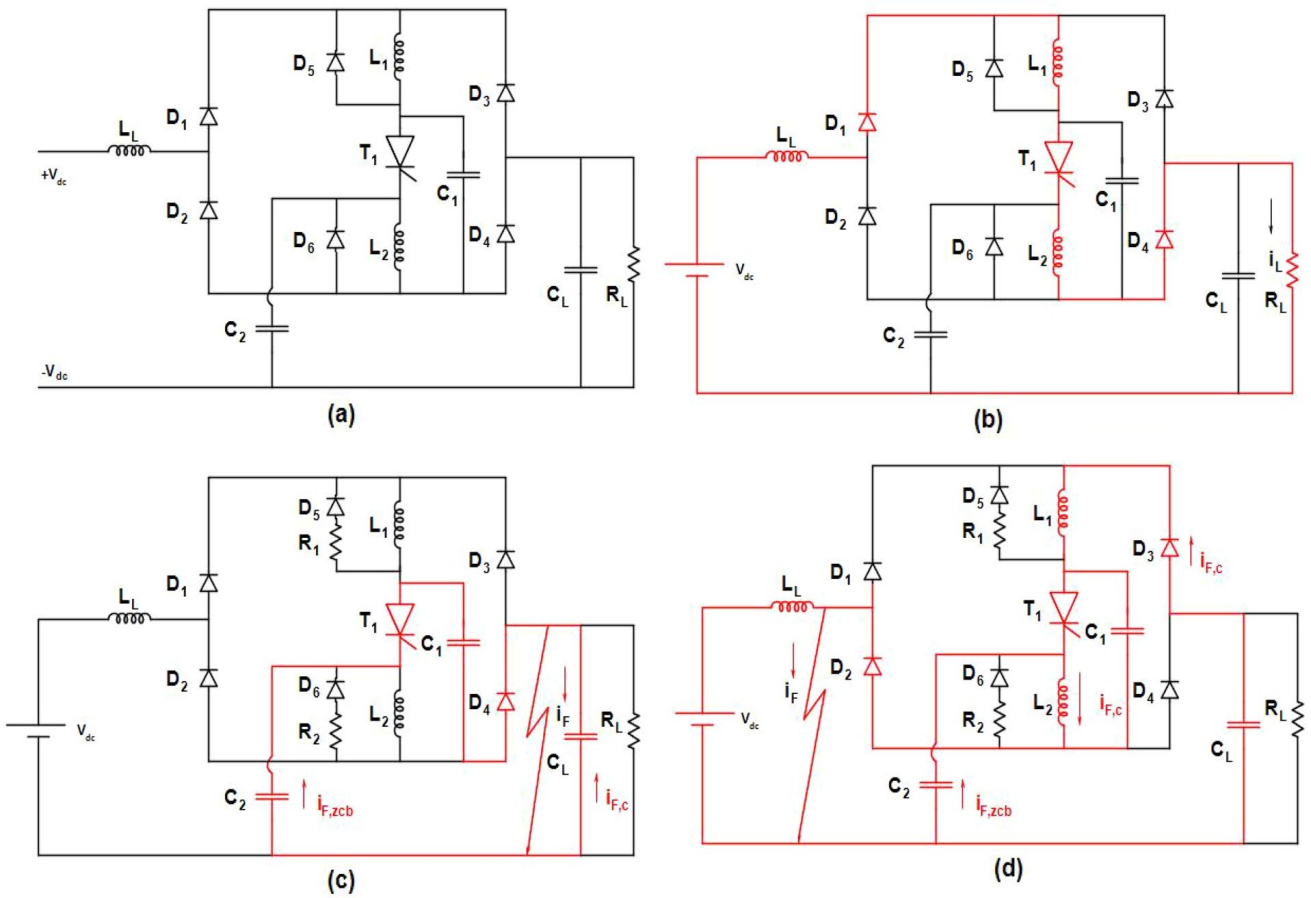
Bidirectional DC circuit breaker modelling and its operating modes.

The modelling of bidirectional DC circuit breaker is depicted in Fig. 1 and the modes of operation are discussed below^23^. In the active state illustrated in (Fig. 1a, b), the inductances of lines L_1_, L_2_, and L_L_ facilitate the flow of I_L_ load current in steady-state, with negligible drop in voltage across the line inductance. Diodes D_1_ and D_4_ conduct during this phase, while D_2_ and D_3_ remain reverse-blocking. Under ideal conditions the voltage across the load will be equal to V_S_, the capacitor C_2_ charges to V_S_ and the capacitor C_1_ discharges accordingly. Upon reversing the connections between the source and load, the circuit breaker (CB) operates similarly. However, D_2_ and D_3_ transition to forward conduction, while D_1_ and D_4_ assume the reverse-blocking state.

In energy storage protection applications, the power flow differs as the existence of bidirectional mode and the precaution is necessary as there is a momentary drop in load current to zero. Hence thyristor functionality should not be affected during the mode transitions.

### Response to load side fault

The fault current follows the indicated red path as illustrated in (Fig. 1c); for a fault occurred at the output. This fault current bifurcates into two components ZCB and C. The former flows in the breaker capacitors, while the latter traverses the load capacitance. Upon fault initiation the Z-Source inductors initially endure abrupt changes in current, thus maintaining their currents at their pre-fault levels denoted by I_L_. Analysing the current at node depicted in Fig. 1c when ZCB increases to I_L_, the current I_T_ becomes zero leading to the commutation of T_1_. Subsequently with T_1_ switched off, two series LC circuits are established: one comprising L_1_, C_1_, and the fault and the other involving L_2_, C_2_, and the fault. Typically with component values within standard ranges both circuits exhibit under-damped resonant responses particularly decoupled when the fault impedance is zero. The opening resonance conditions are contingent upon the steady-state operating point of the circuit.

As these circuits enter resonance the voltage V_C2_ declines while v_C1_ ascends. Upon V_C1_ surpassing source voltage the inductor voltages dip below zero and are damped by diodes D5 and D6. This action restricts the thyristor voltage from exceeding the source voltage, thereby reducing the forward-blocking voltage requisite of T_1_ to V_S_. The inductor current reaches the current peak of the LC circuit, thereby dissipating the stored energy in the damping diodes (D_5_ & D6). In practical implementation a resistor could be introduced in series with the damping diode as depicted in Fig. 1c, to expedite the dissipation of stored energy. However, the voltage drop across the resistor amplifies the blocking state of the thyristor. During resonance the output diode (D_2_ or D_4_) current amounts to the sum of current flowing in two LC series circuits. Conversely, the current in the input diode (D_1_ or D_3_) corresponds solely to that of one LC series by constituting majorly to the output diode current. Notably the time taken by the diode for reverse recovery has no impact the operation of the CB therefore diodes with losses are deemed appropriate. Moreover, the forward voltage drop across a diode typically remains lower than an equivalent thyristor.

### Response to source side faults

The proposed circuit breaker (CB) is equipped to individually handle faults occurring at either of the input of bidirectional current flow. This feature enables swift and independent isolation of faulty lines from the source and load. When the breaker is commutated to be in on-state and a fault occurs across the input a portion of the fault current is drawn from the Z-Source capacitors initially. The behaviour of other passive components varies depending on the load capacitance, as outlined below.

#### RC load

In scenarios where a short circuit fault emerges with existing load capacitance, the fault current follows the pattern illustrated in Fig. 1d. Initially the fault current comprises If_ZCB_, i_fc_, and i_fS_. Under these conditions the collapsing input voltage triggers the deactivation of D1 and D4 while D3 and D2 activate. The fault current originating from the DC source (i_fs_) is constrained to the line inductance I_L_.

Similarly the fault current (i_fc_) sourced from CL is limited to line inductance by the inductors and also the capacitance connected to the Z-Source contributes to the fault current of ZCB. Monitoring the cathode current of T_1_ when i_1_ equals I_L_ (the current passing through L_2_), T_1_’s current decreases to zero and leading to its commutation off. Subsequently, upon T_1_'s deactivation two LC resonant circuits connected in series are established as one involving C_2_, L_2_, and the fault and the other comprising C_1_, C_L_, L_1_, and the fault. These circuits decouple when the fault impedance becomes zero.

The conditions for resonance are dictated by the steady-state operating point of the circuit, with a response analogous to that described in Subsection 3.2 for an output fault. However, in this scenario the load capacitance influences the reaction of one LC circuit. The proposed circuit breaker (CB) is equipped to autonomously handle faults occurring at its source irrespective of the primary direction of current flow. This feature enables swift and independent isolation of faulty lines from a DC power system. And when the breaker is in operational state and a fault occurs across its source terminals, a portion of the initial fault current is drawn from the Z-Source capacitors.

#### Resistive load

When a short circuit fault arises with C_L_ = 0 µF the opening fault current consists of if_ZCB_ and if_S_, here the voltage at both the source and load side collapses immediately. The opening fault current provided by the DC source (i_fs_) is capped at I_L_ by the line inductance L_L_. Consequently, the Z-Source capacitance supplies current to the fault and the load, effectively perceiving the fault current with the load resistance in parallel. Hence, load resistance can be disregarded when the fault manifests as a short circuit. The thyristor current is driven to zero by if_ZCB_ causing its deactivation. After T_1_'s deactivation the current in L_1_ is damped by D_5_ forming a LC resonant circuit in series through C_2_, L_2_, and the fault. However, in this instance a single LC resonant circuit is present.

**Figure 2:**
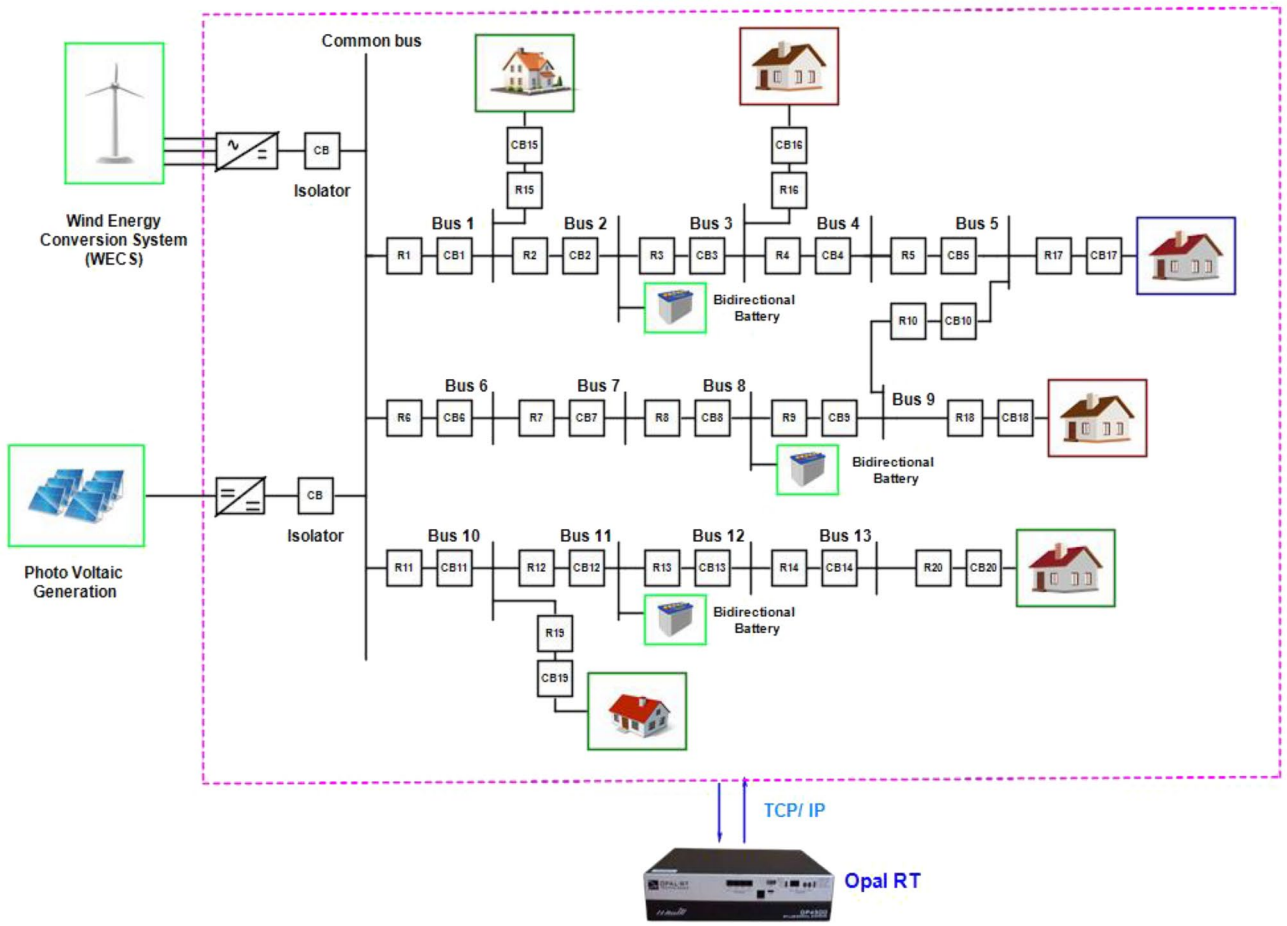
DC microgrid interfaced with real time simulator.

**Figure 3:**
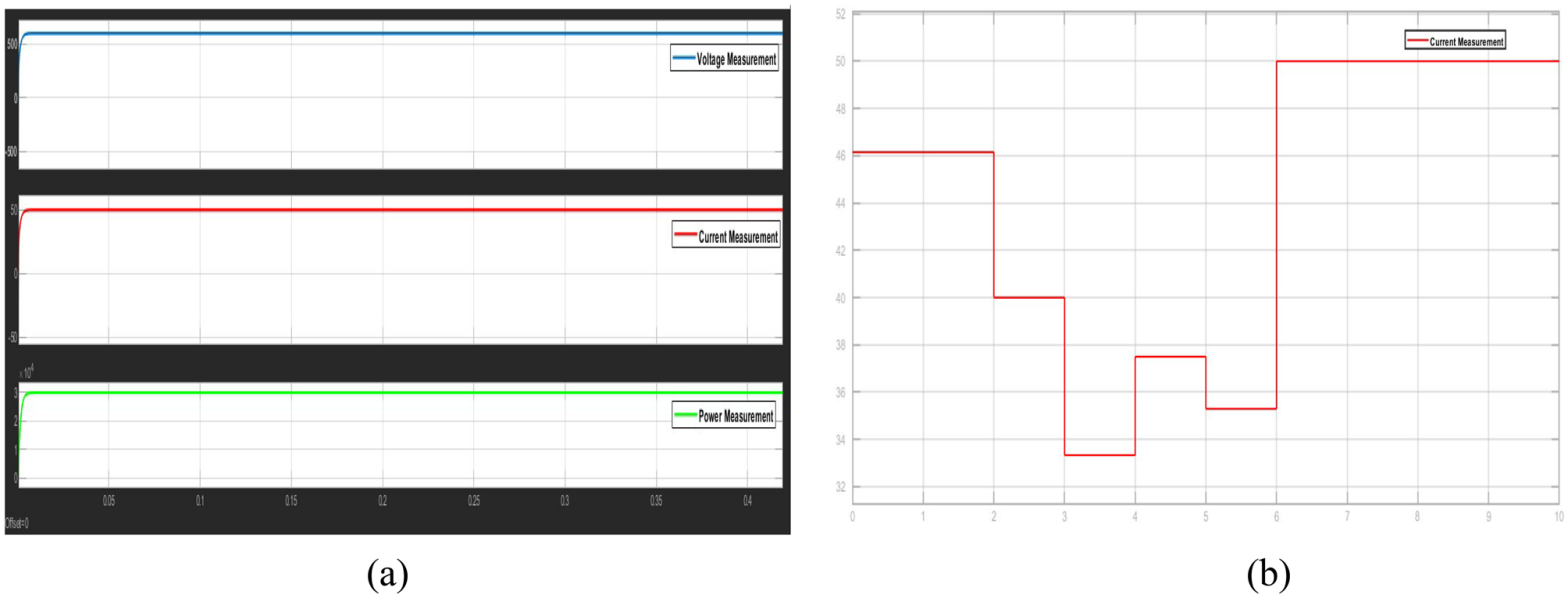
(**a**) Voltage, current and power profile of DC microgrid system, (**b**). Breaker performance under step change in load condition.

Figure 2 illustrates the DC microgrid modelling interfaced with PV, PMSG based wind generation, battery and bidirectional DC circuit breaker. This overall modelling is interfaced with real time simulator OP4500 to test and analyze the protection strategy in real time platform. Figure 3a portrays the grid voltage, grid current and grid power of the DC microgrid rated of 600 V, 50A and 30KW modelled for testing the proposed algorithm. The proposed protection strategy involved with the Z- source breaker in the given 13 bus DC microgrid system is proven to be stable for step load variation in the given system. From the Fig. 3b it is evident that the load current varies for a step variation of load resistance in seconds is not treated as fault and it does not affect the performance of the breaker, thus the breaker is able to clearly identify the difference from fault current and a step load variations ideally.

## Fault detection using graph algorithms

**Figure 4:**
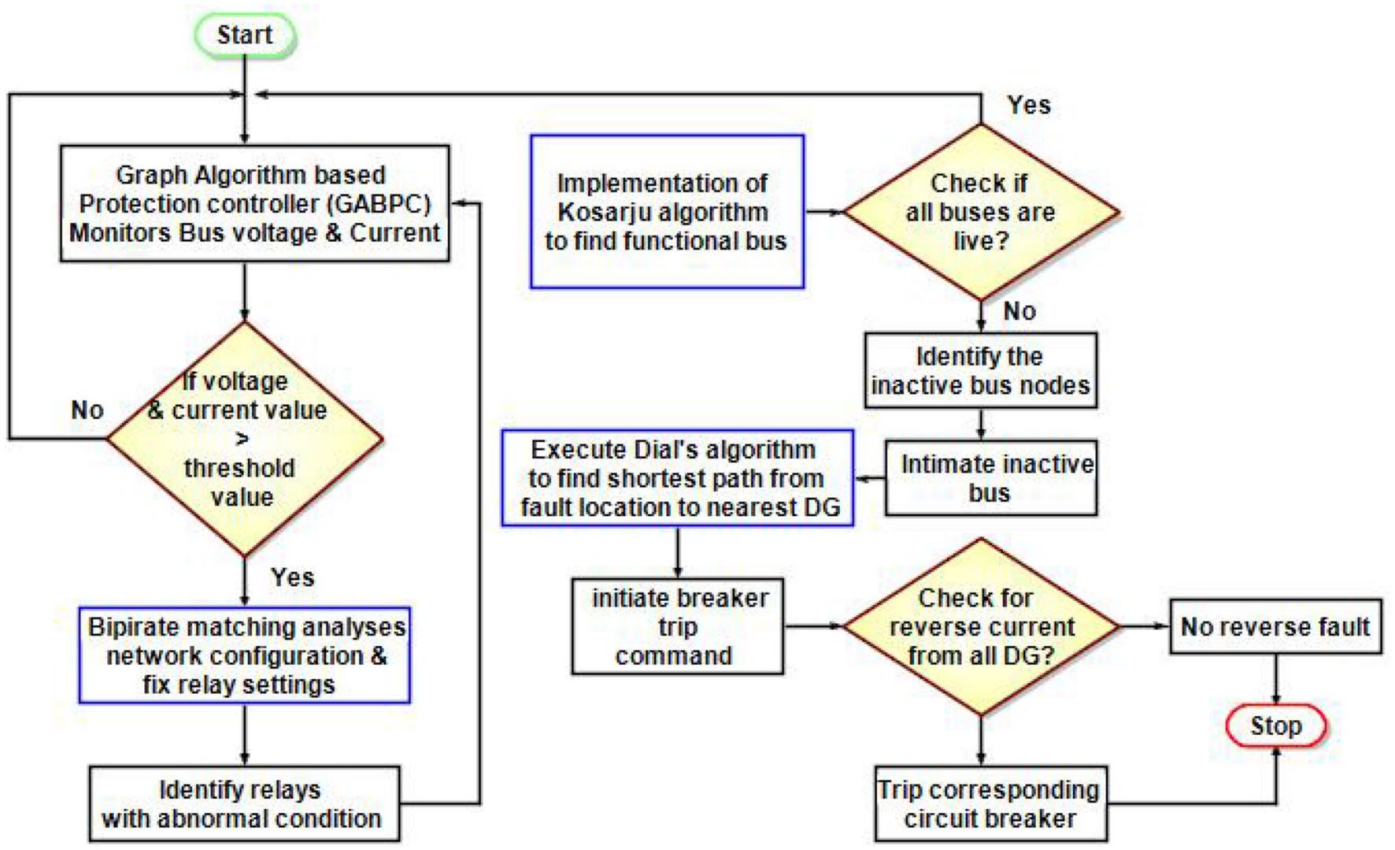
Functionality of the proposed algorithm in DC microgrid.

As illustrated in Fig. 4 the functioning of 13 bus DC microgrid system is handled by Graph Algorithm Based Protection Controller (GABPC). The GABPC continuously examines the line voltage, line current, bus voltage, bus current and power output from different renewables in the DC microgrid system. Following the conventional approach for overcurrent protection, the algorithm identifies the faulted bus if the line voltage, line current, and bus current exceed the fixed threshold limit. The 13 bus DC microgrid system works based on three algorithms, the first and foremost algorithm is bipartite matching. This algorithm finds the existing configuration and works adaptively by varying its configuration based on demands and necessities. The variation in demand leads to change the configuration from ring to radial or radial to ring to support load sharing.

The bipartite matching works as joint or disjoint sets in a graph/network. If the configuration is ring it works based on joint sets and if the configuration is radial it works based on disjoint sets. Notably, the relay settings are monitored and fixed by GABPC considering the voltage, current and power values with a minima and maxima limit of ± 12% of the rated values. On continuation to Bipartite matching, the Kosaraju’s algorithm can be implemented to protect a DC microgrid system, which monitors and determines the functional and disengaged buses. The Kosaraju’s algorithm determines the strongly connected components (SCCs) which are the subset of the vertices in a directed graph and the functional bus nodes in the case of a microgrid. Firstly by performing a Depth-First Search (DFS) in the microgrid system, the algorithm starts with a random starting point and records the end vertices after a complete search. Before performing a second DFS the algorithm transposes the original graph by reversing the direction of all the edges. Thus the proposed adaptive protection system can identify a reverse fault current. In the second DFS the starting vertices are chosen in a decreasing fashion of the earlier pass and thus the disengaged node is determined from the strongest connected node. If a faulted node is identified in the process, the Dial’s algorithm will step on to determine the shortest path from the faulted node to DG. The Dial’s algorithm analyses the shortest path from the faulted node to the nearest DG and initiates the corresponding relay to trip the bidirectional DC circuit breaker. Thus the DG connected near the faulted node is secured prior within the shortest period.

### Performance of the algorithm to fault response

- Step 1: Initially the algorithm monitors bus voltage and current through GABPC.
- Step 2: The bipartite and Kosaraju algorithms work simultaneously to analyse the network configuration of functional and inactive buses. They fix the relay threshold by stacking the values.
- Step 3: The bipartite matching analyses the configuration initially and determines whether it is a ring or radial configuration.
- Step 4: The relay settings are monitored and adjusted by GABPC using bipartite matching, considering the voltage, current, and power values within a minimum and maximum limit of ± 12% of the rated values.
- Step 5: If any abnormalities are found in the stored values, the Kosaraju algorithm immediately tests the functionality of the buses.
- Step 6: Continuing from the Kosaraju algorithm, in finding the inactive bus, the current location of the fault is identified.
- Step 7: Then, GABPC orders the dial's algorithm to find the shortest path from the faulted point to the nearest presence of renewables.
- Step 8: The importance of tripping the breaker nearest to the fault and closest to the renewable source is determined and the corresponding breaker is tripped off.
- Step 9: Once again, it is tested for reverse fault, and if a reverse fault is found the corresponding breaker is immediately tripped off.

## Implementation of thirteen bus DC microgrid *in real* time simulation

Prior to engaging in real-time Hardware-in-the-Loop (HIL) testing for a specific system, Software-in-the-Loop (SIL) analysis is conducted. SIL offers a significant advantage by not necessitating peripheral devices, thereby securing the consistency of the signal. Within this segment, mutually the controller and the plant undergo testing utilizing a real-time simulator (OP4500) constructed on the RT-Lab platform.

**Figure 5:**
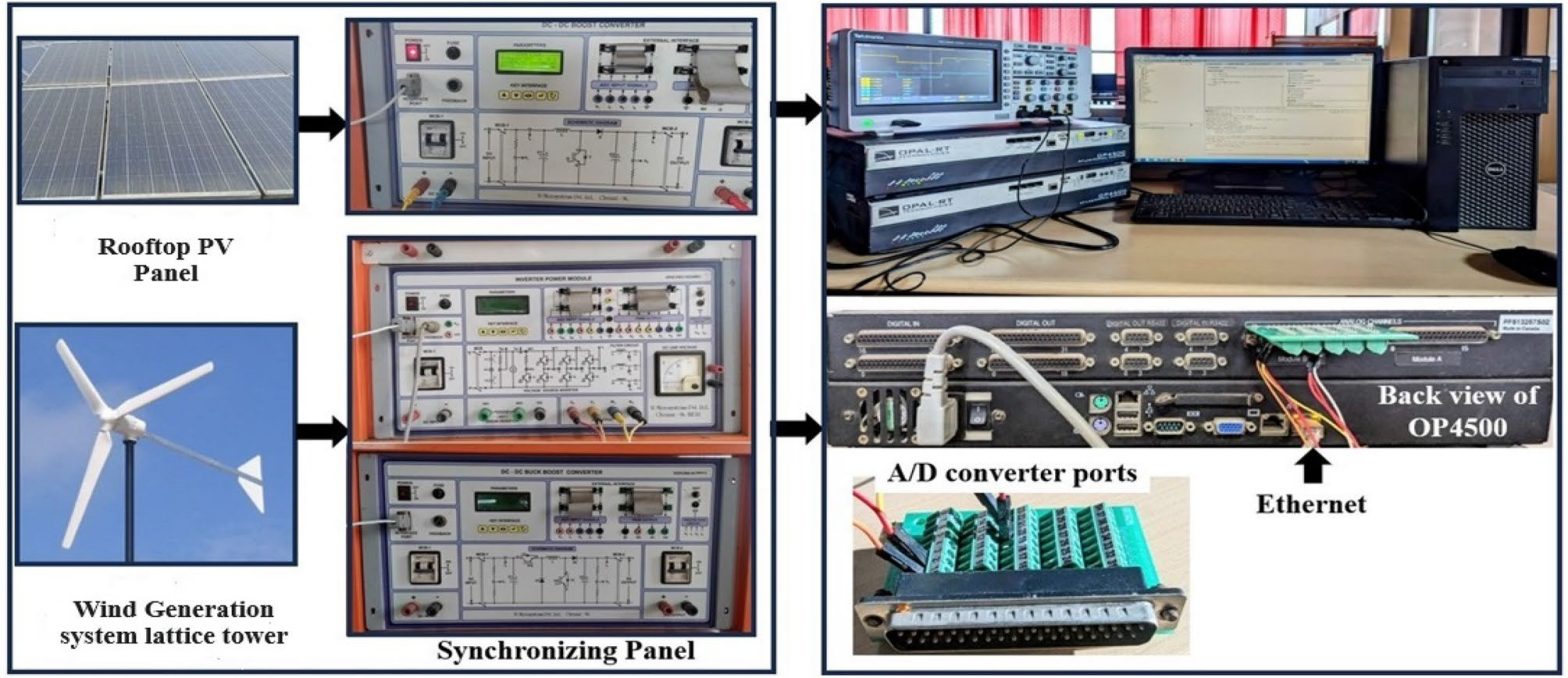
13- bus DC microgrid interfaced with Real time controller for Software in loop (SIL) testing.

Based on the real time data’s collected from the PV, Wind, battery and synchronizing panel installed in smart grid laboratory, VIT Chennai, the Simulink models are modelled and integrated with RT blocks and are accessible on the host computer. This host computer is linked to the RT simulation target via a Transmission Control Protocol (TCP)/Internet Protocol (IP) communication network, as illustrated in Fig. 5. To analyse the system dynamics, various fault scenarios such as line-to-line and line-to-ground faults are tested to validate the performance of the proposed algorithm. Furthermore, the impact of different fault types is also evaluated. Real-time data from the panels are transmitted to the Opal RT processor to facilitate precise real-time analysis. And in Fig. 5 the connectivity between the Opal RT simulator and the host PC is illustrated.

## Results and discussion

**Figure 6:**
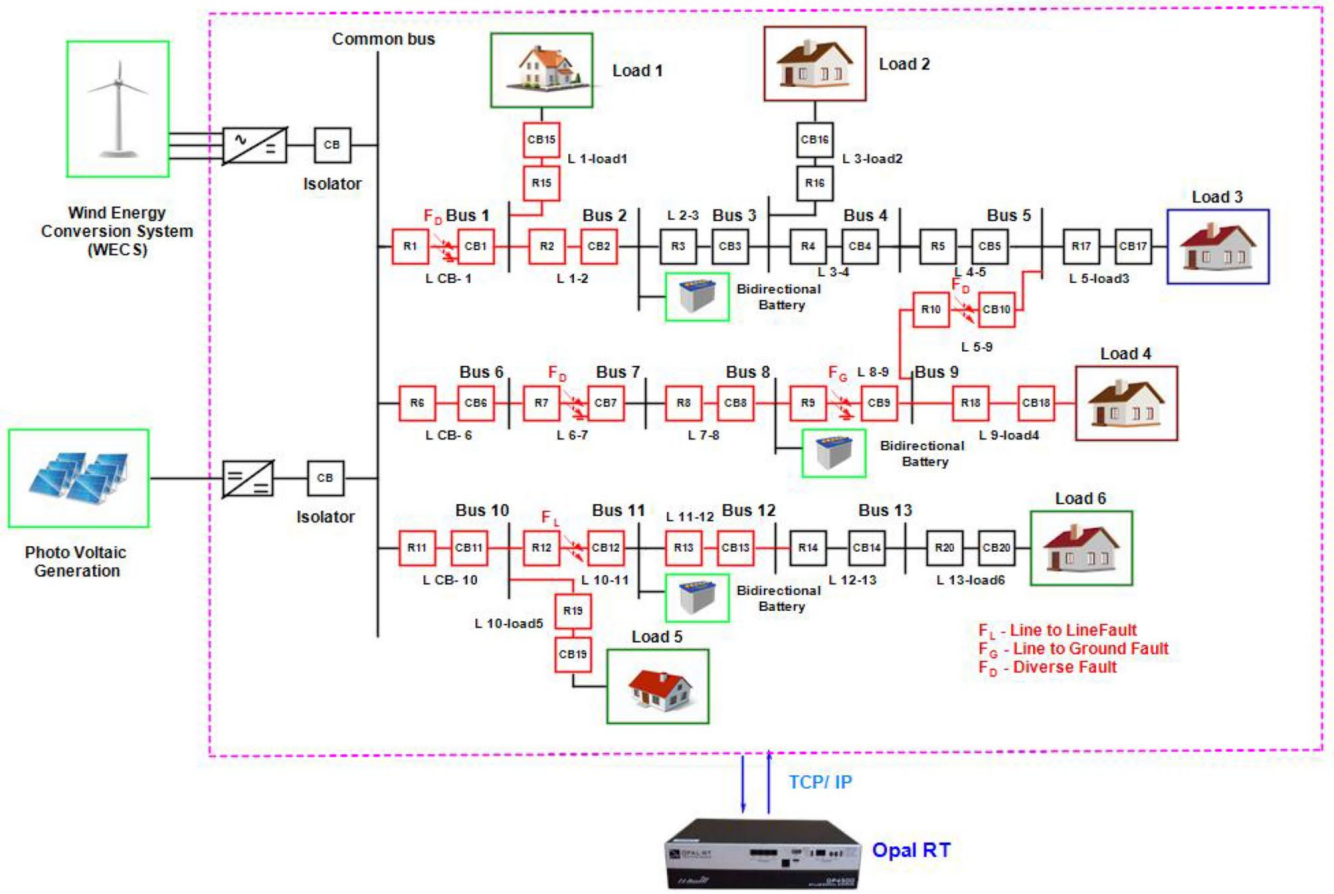
Fault analysis of thirteen bus DC microgrid.

The results are verified in a 13 bus DC microgrid system built in MATLAB Simulink platform integrated with Opal real time (RT) simulator, and the GABPC is interfaced using python program. The proposed graph algorithm based protection strategy is tested and verified in Opal-RT Software in loop (SIL) testing which acts as a real time plant using FPGA processor, and the efficiency of the algorithm is analysed for various operating conditions.. The voltage and current profile of each scenarios is visualized in Digital storage oscilloscope (DSO). During normal and abnormal operating conditions, the bipartite algorithm noted all the parameters and stores in a heap. If the heaped values found abnormal, immediately the information on configuration and signals regarding the abnormal information is sent to kosaraju algorithm by GABPC to find status of active and inactive buses. Furthermore, the signal to find the minimal distance from the current inactive bus to the nearest renewable is investigated by Dial’s algorithm through GABPC. Thus, the shortest path and precise fault location is identified and the corresponding circuit breaker is tripped. This action interrupts the fault and additionally checks for reverse faults. The system ensures fault interruption and verification for reverse fault conditions. Figure 6, shows the various fault tested in the 13 bus DC microgrid system.

### Evaluation of the proposed system under line-to-line fault

**Figure 7:**
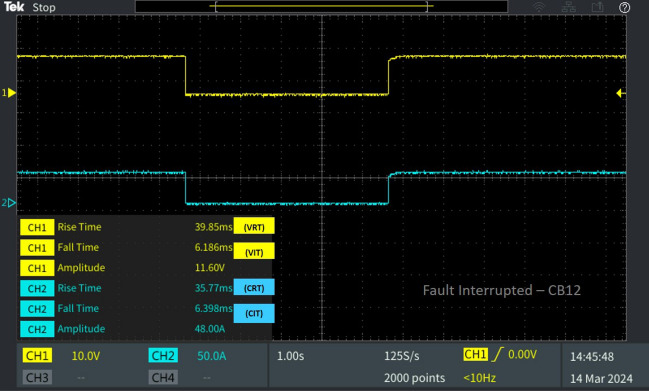
Software in loop test results of CB_12_ interrupting and reclosing during line–line fault.

**Figure 8:**
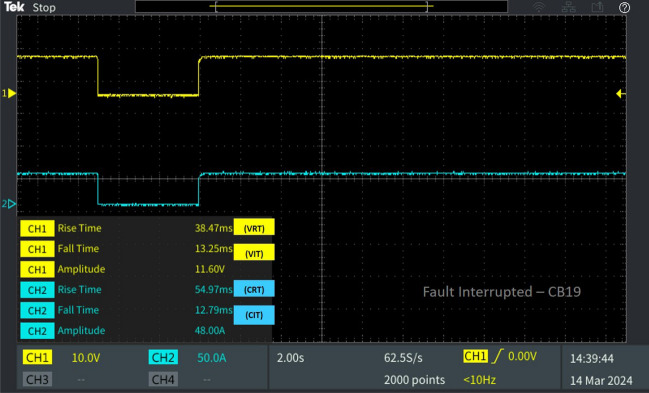
Software in loop test results of CB_19_ interrupting and reclosing during line–line fault.

**Figure 9:**
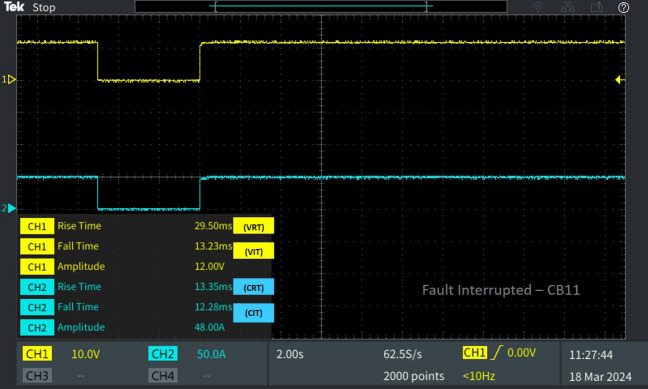
Software in loop test results of CB_11_ interrupting and reclosing during line–line fault.

A line-to-line fault (F_L_) is simulated to test the real-time performance of the system, where a fault is applied at line L_10-11_ for a period of 5 s. The proposed GABPC acts promptly by matching the parameters of the functional nodes in the system using bipartite algorithm, and locates the disengaged nodes using the kosaraju’s algorithm in the system. And by analysing the shortest path and their closeness to the DG source, the relay tripping signals are initiated. Thus the GABPC interrupts the fault by initiating the corresponding breakers near the faulted line using relay coordination. Figures 7–9 show that the algorithm’s implementation proved its efficacy in fault interruption.

The fault occurred on line L_10-11_ and made an impact on the adjacent lines connected to the bus 10 and 11. The difference in voltage and current at the instant of fault is measured by the relays R_11_, R_12_, R_13_ and R_19_ connected adjacent to the fault locations using bipartite algorithm. The impact of the fault is recoiled with the initializing of a tripping signal of CB_12_ by the relay R_12_ located in L_10-11_, as described in Fig. 6.

Figure 8 shows that the proposed adaptive protection system can be evaluated from the operation of CB_12_. For the compatibility of the proposed system in OpalRT, the system voltage range 600 V is made delineate to 12 V considering the gain of 50, and the current rating is maintained at 50A. The breaker CB_12_ interrupts the line–line fault applied for a period of 5 s where the existing grid voltage and load current were 580 V and 48A respectively. The voltage interruption time (V_IT_) and fault current interruption time (C_IT_) of breaker are noted as 6.186 ms 6.398 ms correspondingly. After the fault clearing time of 5 s, the breaker CB_12_ starts to reclose. The breaker CB_12_ recloses with a voltage reclosing time (V_RT_) of 39.85 ms and current reclosing time (C_RT_) of 35.77 ms as mentioned in Table 2.

**Table 2:** Behaviour of the 13 bus system circuit breakers under different fault conditions.

Type of fault		Fault location	Corresponding breaker	V_RT_ (ms)	V_IT_ (ms)	C_RT_ (ms)	C_IT_ (ms)
L-L fault F_L_		Fault at line L_10-11_	CB_12_	39.85	6.186	35.77	6.398
			CB_11_	29.50	13.23	13.5	12.28
			CB_19_	38.47	13.25	54.97	12.79
L-G fault F_G_		Fault at line L_8-9_	CB_9_	30.23	6.614	49.87	6.655
			CB_18_	23.04	6.399	27.01	6.398
Diverse fault F_D_	(L-G)	Fault at line L_CB-1_	CB_1_	23.04	6.399	27.01	6.398
			CB1 reverse fault	13.71	12.58	12.29	12.79
			CB_15_	46.60	12.38	23.15	12.79
	(L-L)	Fault at line L_5-9_	CB_10_	39.85	6.186	35.77	6.398
	(L-G)	Fault at line L_6-7_	CB_7_	22.78	6.399	43.68	6.4

Concurrently, CB19 is interrupted due to a fault occurring in the neighbouring line, consequently leading to the activation of the trip signal by relay R19. Therefore, V_IT_ and C_IT_ of the corresponding circuit breaker CB_19_ were 13.25 ms 12.79 ms as portrayed in Fig. 8. After fault clearance, the breaker recloses at V_RT_ 38.47 ms and C_RT_ of 54.97 with a delay from CB_12_ due to the sequential operation. And the breaker CB_11_ connected neighbouring to the line L_10-11_, isolates from the fault by tripping the breaker with a fault interruption time V_IT_ 13.23 ms and C_IT_ 12.28 ms as displayed in Fig. 9. After fault clearance, the breaker recloses at V_RT_ 29.50 ms and C_RT_ of 13.23 ms comparatively earlier than CB_12._

### Evaluation of the proposed system under line-to-ground fault

**Figure 10:**
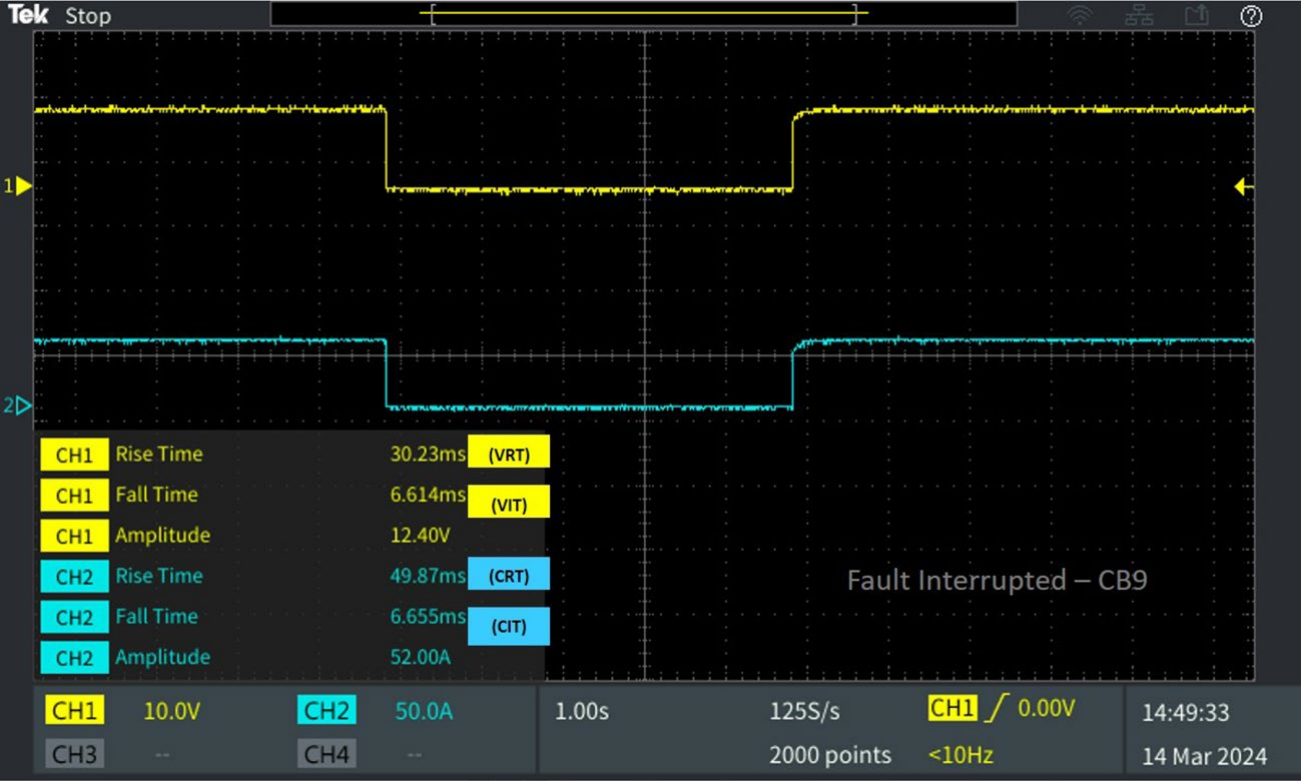
Software in loop test results of CB_9_ interrupting and reclosing during line–ground fault.

**Figure 11:**
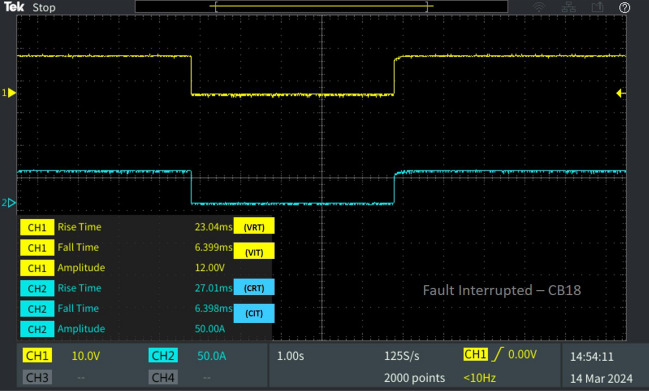
Software in loop test results of CB_18_ interrupting and reclosing during line–ground fault.

A Line-to-Ground fault (F_G_) is simulated between lines L_8-9_ for a duration of 5 s to verify the real-time performance of the proposed protection strategy. The circuit breakers positioned near the fault location trip following the relay signals initiated by the GABPC algorithm, activating relays R_9_, R_8_, R_10_, and R_18_ to trip their corresponding breakers. The performance of CB_9_ situated near the fault and CB_18_ located nearby is compared in Figs. 10, 11. In Fig. 10, the grid voltage of 620 V and load current of 52A is observed on breaker CB9. Upon the occurrence of a Line-to-Ground fault F_G_ on line L_8-9_, CB_9_ trips with an interruption time of V_IT_ 6.614 ms and C_IT_ 6.655 ms from the moment of fault inception. Following fault clearance, CB_9_ recloses with a reclosing time (V_RT_) of 30.23 ms and a reclosing current time (C_RT_) of 49.87 ms.

**Figure 12:**
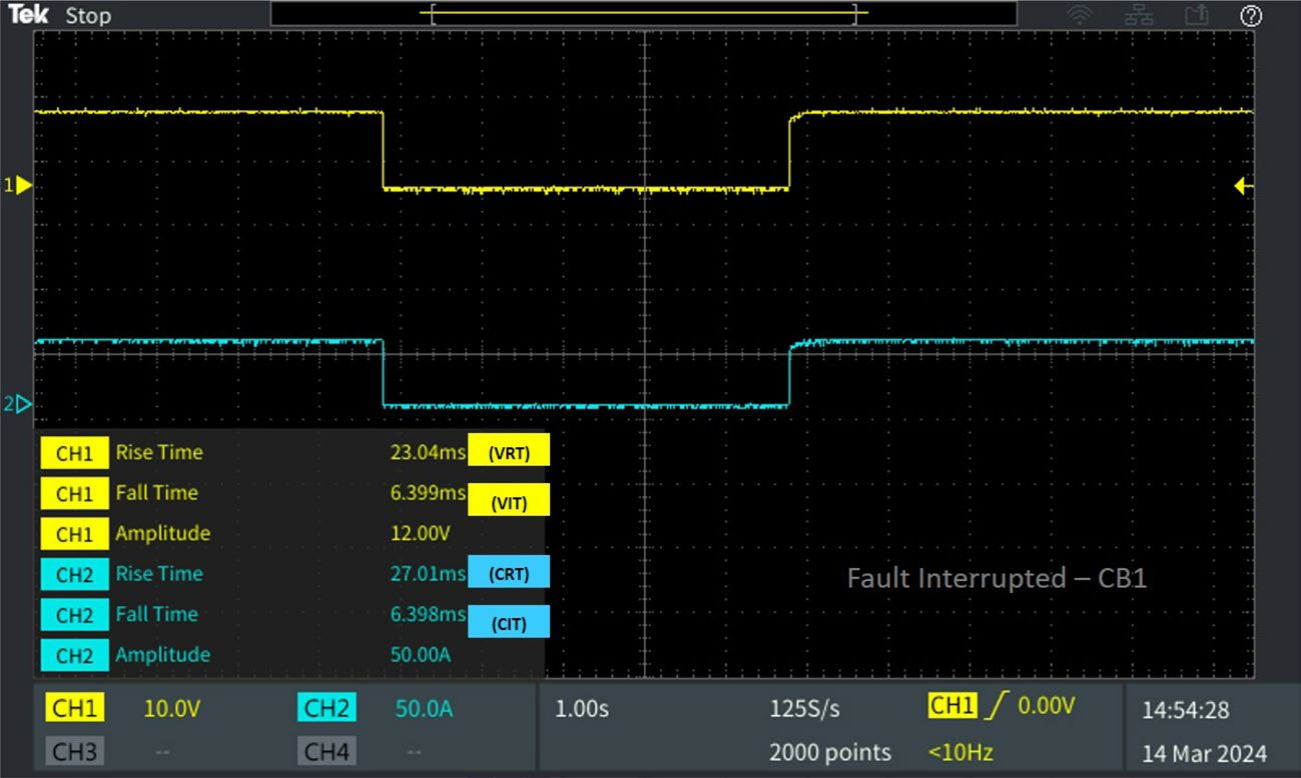
Software in loop test results of CB_1_ interrupting and reclosing during diverse fault.

Concurrently, at the moment of fault occurrence on L_8-9_, load 4 connected to bus 9 is susceptible to the fault. Similarly, breaker CB_18_, positioned between bus 9 and load 4, reacts by interrupting the fault. CB_18_'s fault interruption occurs at a difference in time from CB_9_'s interruption time, with V_IT_ recorded at 6.399 ms and C_IT_ at 6.398 ms as illustrated in Fig. 11. Upon reclosing, CB_18_ achieves a reclosing time of V_RT_ 23.04 ms and C_RT_ 27.01 ms as given in Fig. 12, indicating a shorter reclosing time compared to CB_9_. This is attributed to CB_18_'s distance from the fault location compared to CB_9_, resulting in a quicker reclosing time.

### Evaluation of the proposed system under diverse faults

The behaviour of the proposed adaptive protection strategy is investigated on a 13-bus system by applying multiple faults at different locations over a certain time period. Various fault scenarios of diverse nature (Diverse fault—F_D_) are analysed to assess the robustness of the proposed strategy. A line-to-line fault lasting 5 s is simulated at the radial connecting bus line L_5-9_, while a line-to-ground fault of the same duration is induced on lines L_6-7_ and L_CB-1_, linking the common bus and line L_1_.

**Figure 13:**
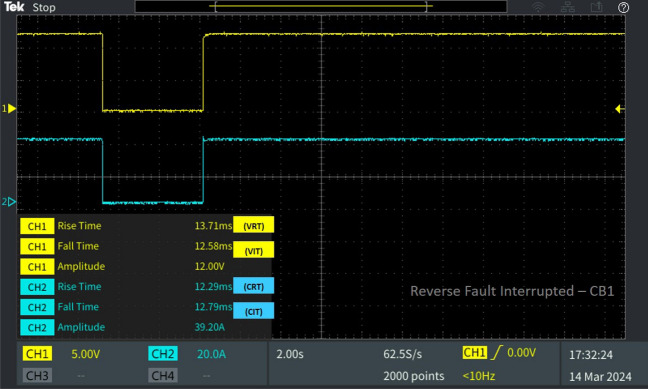
Software in loop test results of CB_1_ interrupting and reclosing during reverse fault current.

Upon detection of the fault at L_CB-1_, Kosaraju’s algorithm identifies disengaged nodes and initiates Dial's algorithm to determine the shortest path from the fault location to the source DG. Relay R1, positioned nearest to the fault location and DG, is consequently activated to trip the circuit breaker CB_1_ by sending a tripping signal. At the occurrence of the fault at L_CB-1_, Kosaraju’s algorithm analyses the situation and triggers the tripping of breaker CB_1_ with an interruption time of V_IT_ 6.399 ms and C_IT_ of 6.398 ms. After fault clearance, the reclosing response time is recorded as V_RT_ 23.04 ms and C_RT_ 27.01 ms as mentioned Table 2. The results from the DSO depicted in Fig. 13 and the results shown in the Table 2, clarifies the proposed protection systems ability to interrupt the reverse fault current prompted by the DG connected near by the breakers location.

**Figure 14:**
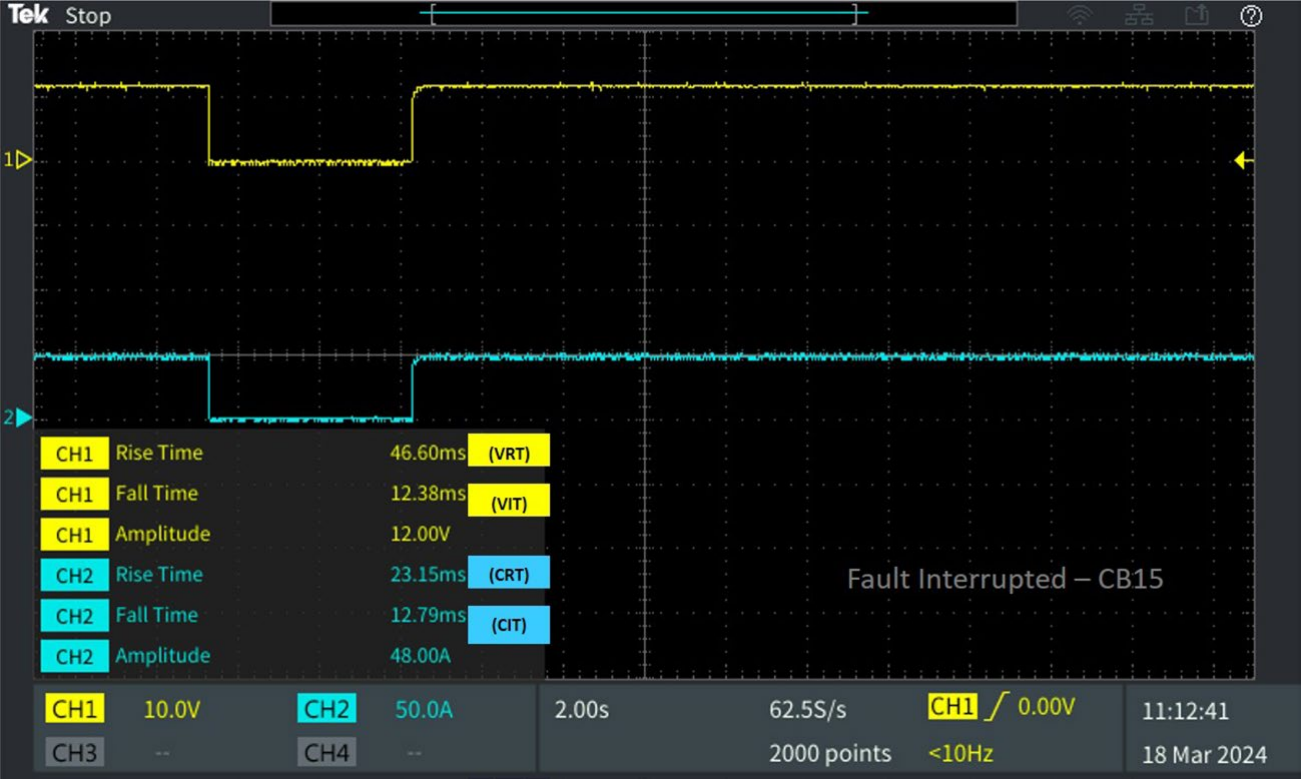
Software in loop test results of CB_15_ interrupting and reclosing during diverse fault.

**Figure 15:**
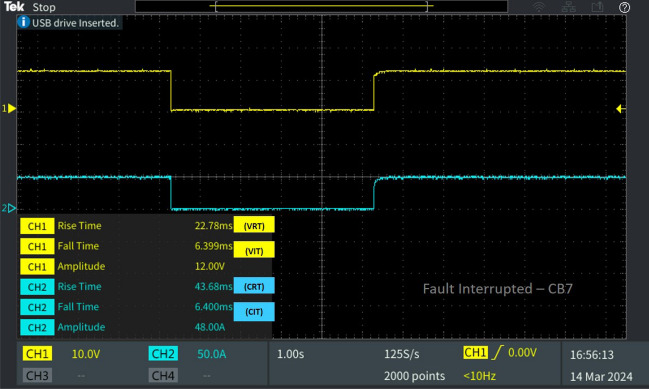
Software in loop test results of CB_7_ interrupting and reclosing during diverse fault.

In Fig. 14 the breaker operation of CB_15_ is depicted for the line–line fault occurred on the line L_5-9_ under diverse fault conditions. For the fault occurred in L_5-9_ the breakers CB_1_ and CB_15_ connected to the load, reacts by interrupting of the fault current. The interruption time V_IT_ and C_IT_ of CB_15_ is 12.38 ms and 12.79 ms, followed by the reclosing time of V_RT_ and C_RT_ of 46.60 ms and 23.15 ms. Concurrently, breaker CB_7_, situated closer to the fault location L_6-7_, is tripped by relay R_7_ at the same instant as CB_1_, with a fault interruption time of V_IT_ 6.399 ms and C_IT_ of 6.4 ms. The time taken for the breaker voltage and current to rise after reclosing is V_RT_ 22.78 ms and C_RT_ of 43.68 ms as depicted in Fig. 15.

**Figure 16:**
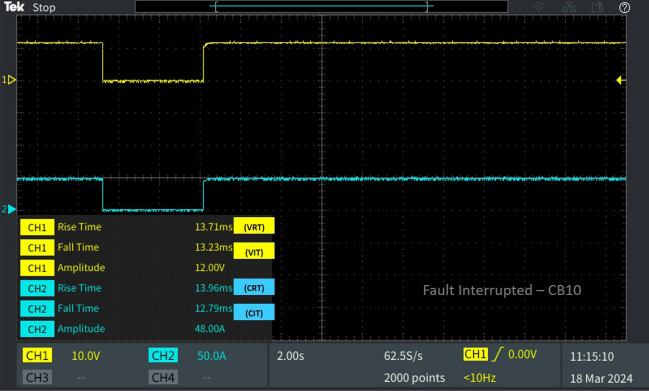
Software in loop test results of CB_10_ interrupting and reclosing during diverse fault.

Similarly, for the fault occurred on the radial connecting line L_5-9_ circuit breaker CB_10_ is tripped for the line-to-line fault, with the interruption time recorded as V_IT_ 6.186 ms and C_IT_ 6.398 ms. The reclosing time is noted as V_RT_ 39.85 ms and C_RT_ 35.77 ms as showed in Fig. 16. Instantaneous faults occurring at multiple locations are successfully interrupted and cleared within minimal time. All breakers in the 13-bus system reclose after fault clearance with no peak in reclosing current, as evidenced by the real-time results. The reduced runtime of the proposed algorithm ensures faster fault detection and response, leading to improved system reliability, reduced downtime, and enhanced overall performance of the DC microgrid. The findings underscore the effectiveness of the proposed algorithm in promptly identifying and addressing faults, making it suitable for practical implementation in real-world scenarios. Its ability to detect simultaneous faults and react instantly to all fault types without delay is particularly advantageous for ensuring the stability and reliability of the DC microgrid system. By swiftly interrupting fault currents and initiating necessary corrective actions, the algorithm minimizes runtime, thereby enhancing the overall performance and efficiency of the microgrid system.

## Conclusion

Incorporating adaptive techniques in existing z-source breaker topology is an emerging trend in DC microgrid protection. The GABPC aids in identifying the functional nodes that operate autonomously and swiftly deactivate the breaker in reaction to a fault. The DCCB verifies the proposed algorithm-based protection strategy in a 600 V/30 kW renewable integrated DC microgrid to validate the system under different fault conditions as given in Table 2 and in future MVDC and HVDC system will be considered. The function of bipartite matching helps to fix the relay threshold and the Kosaraju algorithm finds the functional and disengaged buses in transmission line. Thereby all the paths are noted from the location of the fault to the nearest operating distributed generation by Dial’s algorithm. Henceforth, this amalgamation of proposed algorithm finds the least distance path from the fault location to the nearby distributed generation. This trips the bidirectional DC circuit breaker interfaced with proposed algorithm and provides the minimal time to detect and interrupt the Line to ground fault, line to line fault and diverse fault conditions.

### Supplementary Information

Supplementary Information.

## Data Availability

The datasets of the voltage and current profile of the relays used in the 13 bus microgrid system under normal condition and fault conditions is given in the supplementary material. For further detail datasets analyzed during the current study is available from the corresponding author (Sumathi V, E-mail: vsumathi@vit.ac.in) on reasonable request.
